# Study on room temperature gas-sensing performance of CuO film-decorated ordered porous ZnO composite by In_2_O_3_ sensitization

**DOI:** 10.1098/rsos.171788

**Published:** 2018-02-14

**Authors:** Tian-tian Li, Na Bao, Ai-fang Geng, Hui Yu, Ying Yang, Xiang-ting Dong

**Affiliations:** School of Chemistry and Environmental Engineering, Changchun University of Science and Technology, Changchun 130022, People's Republic of China

**Keywords:** ordered mesoporous ZnO, electroplating after chemical plating method, CuO film, In_2_O_3_, NO*_x_*, gas sensor

## Abstract

For the first time, ordered mesoporous ZnO nanoparticles have been synthesized by a template method. The electroplating after chemical plating method was creatively used to form copper film on the surface of the prepared ZnO, and then a CuO film-decorated ordered porous ZnO composite (CuO/ZnO) was obtained by a high-temperature oxidation method. In_2_O_3_ was loaded into the prepared CuO film–ZnO by an ultrasonic-assisted method to sensitize the room temperature gas-sensing performance of the prepared CuO/ZnO materials. The doped In_2_O_3_ could effectively improve the gas-sensing properties of the prepared materials to nitrogen oxides (NO*_x_*) at room temperature. The 1% In_2_O_3_ doped CuO/ZnO sample (1 wt% In_2_O_3_–CuO/ZnO) showed the best gas-sensing properties whose response to 100 ppm NO*_x_* reached 82%, and the detectable minimum concentration reached 1 ppm at room temperature. The prepared materials had a good selectivity, better response, very low detection limit, and high sensitivity to NO*_x_* gas at room temperature, which would have a great development space in the gas sensor field and a great research value.

## 1. Introduction

In recent years, environmental monitoring gas sensors have attracted great attention. Gas sensors can detect various inflammable, explosive, toxic, and harmful gases accurately and effectively. The development of efficient, low cost, highly sensitive, highly selective and convenient chemical sensors can precisely recognize and measure the presence of toxic gases and human exposure levels, which have become a significant endeavour for the well-being and welfare of people, safety and process control in industrial applications [1]. As gas-sensing materials, metal oxide semiconductors (MOS) have promising applications in monitoring air pollution, detecting toxic/explosive gases etc. which have been widely investigated, and have become a hotspot [2,3]. The metal oxide-based gas sensors are used widely for the detection of toxic gaseous species not only because of their superior thermal and physical stability but also due to their ability to detect very low gas concentrations [4]. Many attempts have been made to enhance the sensitivity or selectivity of oxide nanomaterials by surface modification, metal-doping, loading and the use of core shell structures [5–7].

Among various kinds of developed oxide gas-sensing materials, ZnO has attracted great attention. ZnO is a functional n-type semiconductor, which has been considered as an essential semiconductor gas-sensing material because of its high chemical stability, suitability to doping, harmlessness and economic nature [8,9]. However, pure ZnO has some inherent disadvantages, including low sensitivity, high working temperature, long response and recovery time etc. which may prevent its further development [10]. Micro/nanostructured MOS have received much attention because they have high sensitivity to small concentration of gases, low fabrication cost, long-term stability and low power consumption [11]. It is known that the morphology has a great influence on the gas-sensing properties of materials, and current researches are focused on obtaining special morphology and structure of ZnO nanomaterials by different means. Lots of different morphological ZnO and ZnO-based composite have been prepared, such as ZnO nanoflakes [12], ZnO nanowires [13], ZnO micro-flowers [14], porous ZnO micro-sheets [15], ZnO-decorated Fe_2_O_3_ [16] and so on.

CuO is a p-type semiconductor with a low band gap of 1.2–2.0 eV, and its p-type characteristic provides a route to form p–n junctions with n-type metal oxides [17]. CuO has been used successfully to detect H_2_S using a range of p–n composite nanostructures [18–20]. Composite type sensor using two or multiple oxide material in single or multiple layers is an effective way to enhance the selectivity of the sensors. Various authors have reported selective and sensitive gas-sensing characteristics of composite oxide material. ZnO and CuO composites have been reported in gas sensing. Combination of one-dimensional ZnO and CuO microstructure may also exhibit enhanced gas-sensing property [18,21]. Rai *et al.* [22] reported the modulation of ZnO nanorod electronic properties through CuO nanoparticles and the use of functionalized ZnO nanorods for CO sensing applications. A wet chemical technique was used to prepare CuO-doped ZnO semiconductor nanomaterials with nearly controlled rod shape structure [23].

The gas-sensing properties of materials are closely related with the density and species of surface adsorptive gases. Therefore, for high performances of gas-sensitive materials they need to have a larger Brunauer--Emmett--Teller (BET) surface area. Late *et al.* [24] presented a comprehensive overview of recent developments in the application of two-dimensional (2D) layered inorganic nanomaterials as sensors, and they did a lot of works in the 2D layered sensor materials field which included the large-area MoS_2_ sheets gas sensor [25], 2D WS_2_-based sensor [26,27], single-layer MoSe_2_-based gas sensor [28], SnSe_2_ nanosheets sensor [29] and 2D black phosphorus sensor [30,31]. Porous materials have a much bigger BET surface area and much stronger gas adsorption ability, which greatly improve the gas-sensing performance of the porous materials. Porous ZnO nano/microstructures have also drawn extensive research attention. Specifically, the introduction of pores into ZnO nano/microstructures facilitates the gas diffusion and mass transport, enormously improving gas sensor performance [32]. Liu *et al*. [33] reported the synthesis of three-dimensional hierarchical ZnO porous structures functionalized by Au. Wang *et al.* [34] reported a facile approach to prepare nest-like three-dimensional porous ZnO with hierarchically 2D lamellar structures. The nest-like ZnO hierarchically porous structures displayed a superior gas-sensing performance. Liu *et al.* [35] reported the synthesis of single-crystalline porous ZnO nanosheets by annealing ZnS (ethylenediamine) complex precursor, which exhibited highly sensitive performance. Huang *et al.* [36] reported tunable macro–mesoporous ZnO (M/m-ZnO) nanostructures, and the prepared M/m-ZnO nanostructures demonstrated better ethanol and acetone sensing properties. Template and template-free mesoporous ZnO-based structures have been widely prepared as gas materials [37].

In this study, ordered mesoporous ZnO nanoparticles have been synthesized by a template method. The electroplating after chemical plating method was creatively used to form copper film on the porous ZnO surface, and then a CuO film-decorated ordered porous ZnO composite (CuO film–ZnO) was obtained by a high-temperature oxidation method. In_2_O_3_ was loaded in the prepared CuO film–ZnO by ultrasonic-assisted method to sensitize the room temperature gas-sensing performance of the prepared materials. The fluffy porous structure was helpful for gas diffusion and surface reaction, which led to the sensor exhibiting faster response. The copper film was more uniform when the electroplating after chemical plating method was used to form copper film, and the n–p heterojunction between CuO film and ZnO matrix played an important role in the enhancement of the sensor response and selectively. The In_2_O_3_ played a sensitizer role to sensitize the surface reaction. In_2_O_3_ was a catalyst, it reduced the activation energy of the oxidation–reduction reaction on the surface of composites, efficiently separated the oxygen molecules, improved the activity of gas-sensitive materials, increased the free electron amount and expedited the reaction rate. As such, the gas sensitivity of the composites was enhanced. The prepared material greatly reduced the working temperature of the sensor, which would have a great development prospect and great research value in the sensor field.

## 2. Experimental

### 2.1. Materials

All chemicals used in our work were of analytical grade, and used without any further purification. Cetyltrimethylammonium bromide (CTMAB, A.R.) was purchased from Changzhou Xinhua Reagent Research Institute in China. Zn(Ac)_2_·2H_2_O was purchased from Tianjin Guangfu Fine Chemical Research Institute in China. Sodium hydroxide (NaOH, A.R.) was purchased from Beijing Chemical Works Research Institute in China. Potassium sodium tartrate tetrahydrate (C_4_H_4_O_6_KNa·4H_2_O, A.R.) was purchased from Tianjin Guangfu Technology Development Co. Ltd in China. Formaldehyde solution (HCHO, A.R.) was purchased from Tianjin Fuchen Chemical Reagents Factory in China. Ethylene diamine tetraacetic acid disodium (EDTA-2Na, A.R.) was purchased from Shenyang Reagent Factory in China. Copper(II) sulfate pentahydrate (CuSO_4_·5H_2_O, A.R.) was purchased from Sinopharm Chemical Reagent Co. Ltd in China. Ethanol and deionized water were used for all processes of washing and dissolution.

### 2.2. Preparation of materials

#### 2.2.1. Preparation of mesoporous ZnO nanoparticles

One gram of CTMAB was added into 480 ml of deionized water, and the mixture was vigorously stirred until the solution was homogeneous at 80°C. About 4.92 g of Zn(Ac)_2_· 2H_2_O was dissolved into the solution with stirring. The pH of the solution was adjusted with LiOH to alkaline; the reaction then proceeded at 80°C for 2 h. The product was filtered, washed with deionized water and dried at room temperature to get the powder sample. The original sample was calcined at 500°C for 4 h, and the ordered mesoporous ZnO material was finally obtained.

#### 2.2.2. Preparation of CuO film on the mesoporous ZnO matrix

The electroless plating solution was prepared according to table 1. The prepared ordered porous ZnO material was added into the electroless plating solution, which was reacted for 1 h under vigorous stirring. The powder was filtered, washed with deionized water and dried to afford a reddish brown powder with metallic lustre.

**Table 1.** Electroless plating solution formula.

reagents	CuSO_4_^.^5H_2_O	HCHO	EDTA-2Na	sodium potassium tartrate	potassium ferrocyanide	dipyridyl	NaOH
concentration	25 g l^−1^	20–40 g l^−1^	20–40 g l^−1^	10–20 g l^−1^	10 mg l^−1^	10 mg l^−1^	20%

The prepared powder was put into semipermeable membrane, and the negative pole was inserted into the powder. The powder was immersed in the plating solution (table 2). The sheet copper was used as anode, voltage was regulated at 17 V, electricity was regulated at 1 A and the plating solution was electrified for 1 h. The powder was filtered, washed with deionized water, dried, calcined at 500°C for 4 h to afford a black CuO film–ZnO powder sample.

**Table 2.** Plating solution formula.

reagents	CuSO_4_^.^5H_2_O	NaOH	citric acid	sodium potassium tartrate	deionized water
amount	13 g	31.25 g	50 g	10 g	250 ml

#### 2.2.3. Preparation of In_2_O_3_ sensitization CuO film–ZnO composite material

In order to study the effect of different In_2_O_3_ decoration amounts on the gas-sensing performance, a series of In_2_O_3_ decorated CuO film–ZnO composites were synthesized. In a typical procedure, 0.1 g prepared CuO film–ZnO product was dispersed in 20 ml deionized water with the aid of ultrasonication. A certain amount of In(NO_3_)_3_ (In(NO_3_)_3_ concentration: 10 wt%, 15 wt%, 20 wt% and 25 wt%) was added in the above solution. The reaction system was dispersed in ultrasonic for 10 min, and the suspension liquid was aged for 2 h at room temperature. The suspension liquid was filtered, washed with deionized water and dried for 10 h at 60°C. The prepared samples were heated for 4 h at 500°C with a warming rate of 2°C min^−1^ to afford the products. The In-loaded contents were obtained with an inductively coupled plasma–optical emission spectrometer (ICAP 6000 Series), and the corresponding doping contents of In_2_O_3_ were calculated which were 0.5, 1, 3 and 6 wt%, respectively.

### 2.3. Characterization

Powder X-ray diffraction (XRD) patterns were collected with a Siemens D5005 diffractometer using Cu–K*α* radiation (*λ* = 1.5418 Å and operating at 30 kV and 20 mA). Transmission electron microscopy (TEM) images were taken with a JEOL 2010 TEM instrument. The scanning election microscopy (SEM) images were taken with a JEOL JSM-5600 L. Physical adsorption of nitrogen was performed with a Micromeritics ASAP2010M volumetric adsorption analyser at 77 K. A sample was degassed in vacuum at 573 K for 12 h before measurement. Surface area was calculated based on the BET model, while pore size was computed using the Barrett–Joyner–Halenda method.

### 2.4. The preparation of gas sensor

**Figure 1.**
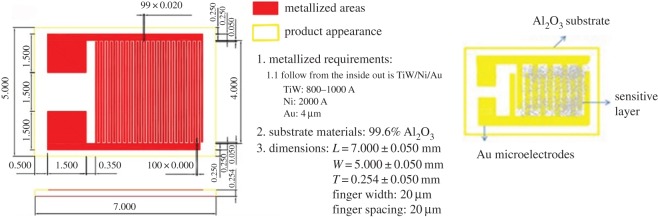
Parameters of interdigitated gold electrode and schematic diagram of the prepared gas sensor.

In this study, an interdigitated Au electrode was selected for the gas-sensing detection. The sample was disposed as follows: about 3 mg of the prepared sample was dispersed in ethanol, which was dispersed ultrasonically for about 20 min. The sample material was spin-coated on the interdigitated electrode surface to form a thin film, which was dried to obtain the gas sensor. The parameters of interdigitated gold electrode and schematic diagram of the prepared gas sensor are shown in figure 1.

The sensor was welded onto a socket, and the electrical properties of the sensor were measured by a JF02E intelligent gas-sensing analysis system. The sensor response was defined as

2.1 $$S(\%)=\left[\frac{\text{( }R_{a}-R_{g}\text{) }}{R_{a}}\right]\times 100\%.$$

Here, *R*_a_ and *R*_g_ were the resistances of the sensors in the air and target gas, respectively. The response and recovery time were defined as the time taken by the sensors to achieve 85% of the total resistance change in the case of adsorption and desorption, respectively [38–40].

## 3. Results and discussion

### 3.1. Structure and morphology

**Figure 2.**
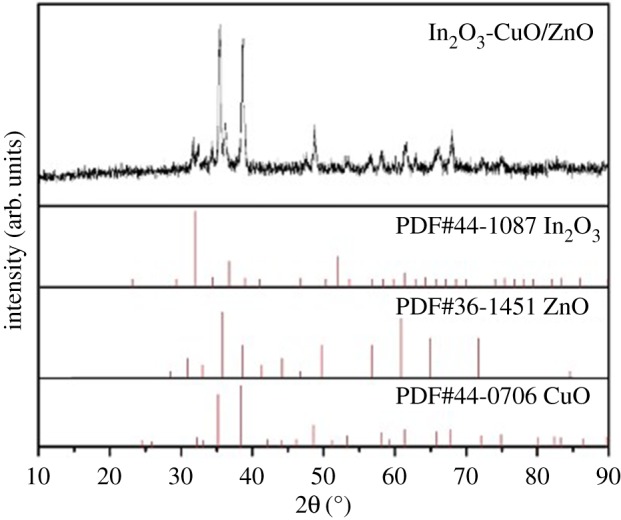
Wide-angle XRD pattern of the 1 wt% In_2_O_3_–CuO/ZnO sample.

Figure 2 shows the wide-angle XRD pattern of the prepared ordered mesoporous 1 wt% In_2_O_3_–CuO/ZnO sample. The sharp diffraction peaks indicated the highly crystalline characteristic of 1 wt% In_2_O_3_–CuO/ZnO samples. All of the diffraction peaks were consistent with the standard card of CuO (JCPDS No. 44-0706), which showed that the CuO has been deposited on the surface of the ZnO. The diffraction peaks of wurtzite ZnO (JCPDS No. 36-1451) could be seen in the prepared material. In the XRD pattern, the characteristic peaks of the In_2_O_3_ could also be seen. It showed that the In_2_O_3_ has been doped into the prepared CuO film–ZnO matrix material.

**Figure 3.**
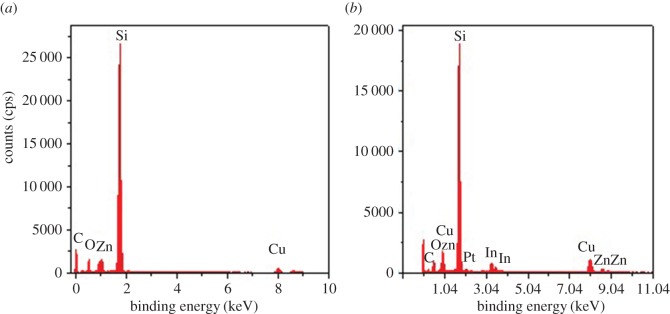
Energy-dispersive X-ray spectra of the (*a*) CuO/ZnO sample and (*b*) 1 wt% In_2_O_3_–CuO/ZnO sample.

Figure 3 shows the energy-dispersive X-ray spectra of CuO/ZnO and 1 wt% In_2_O_3_–CuO/ZnO samples. From figure 3*a*, the peaks of C, Zn, O, Si and Cu could be seen in CuO/ZnO sample. The sample was first placed on a silicon wafer when the sample was tested, which led to the highest Si content peak. The C and O were mainly from the conducting resin. In figure 3*b*, the indium (In) content peaks could be seen in 1 wt% In_2_O_3_–CuO/ZnO sample, which could prove the existence of the In_2_O_3_ in 1 wt% In_2_O_3_–CuO/ZnO sample, and the In_2_O_3_ has been successfully assembled into the CuO film–ZnO matrix. The Pt content peak was attributed to the Pt conductive agent sprayed on the surface of the sample. There were no peaks of other elements in samples, which indicated that the prepared samples were pure.

**Figure 4.**
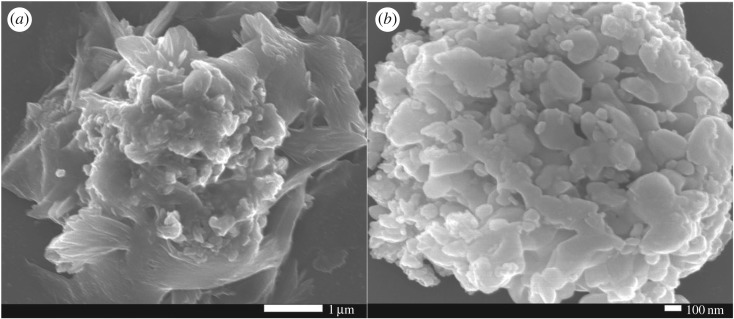
SEM images of the (*a*) CuO/ZnO and (*b*) 1% In_2_O_3_–CuO/ZnO sample.

Figure 4 shows the SEM image of CuO film–ZnO and 1 wt% In_2_O_3_–CuO/ZnO samples. From figure 4*a*, the CuO/ZnO sample showed a whole flower-like appearance, whose surface was smooth. From figure 4*b*, the 1 wt% In_2_O_3_–CuO/ZnO sample showed an analogous flower-like structure, which obtained from the petals of flower-like CuO/ZnO sample fallen off, the analogous flower-like structures were heaped with irregular flaky grain.

**Figure 5.**
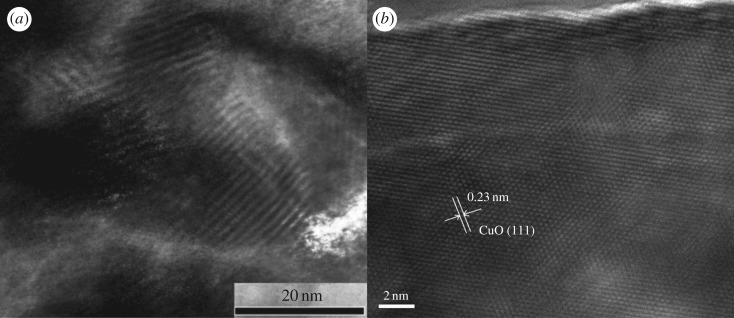
(*a*) TEM image and (*b*) HRTEM image of the 1 wt% In_2_O_3_–CuO/ZnO sample.

Figure 5*a* shows the TEM image of the 1 wt% In_2_O_3_–CuO/ZnO sample, which was taken parallel to the direction of the channels. From figure 5*a*, it could be seen that there were a lot of ordered pores in the sample. Figure 5*b* shows the high-resolution TEM (HRTEM) image of the 1% In_2_O_3_–CuO/ZnO sample. From figure 5*b*, it could be seen that the lattice fringes of the sample were very clear, which were the lattice fringes of CuO. In the HRTEM images, the ordered pores could be very clearly seen under the CuO lattice fringes, which indicated that the CuO thin films were formed on the surface of the prepared sample.

### 3.2. Gas-sensing performance

**Figure 6.**
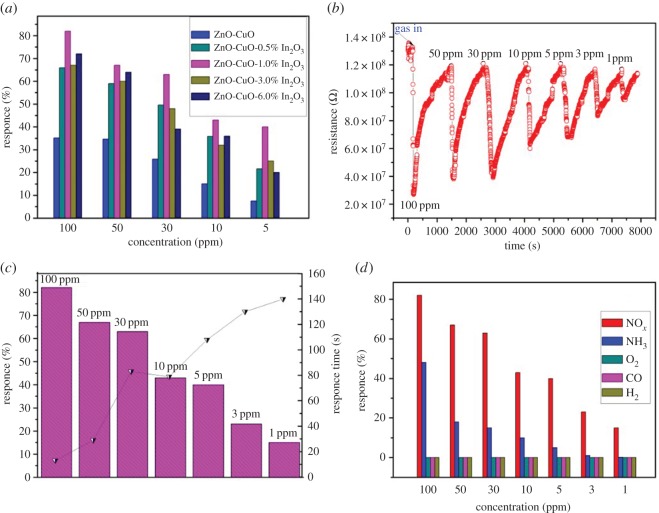
(*a*) Gas response bar charts of different In_2_O_3_-doped concentration samples to different concentrations of NO*_x_*; (*b*) dynamic response–recovery curves of 1 wt% In_2_O_3_–CuO/ZnO sample; (*c*) response and response time curve of the 1 wt% In_2_O_3_–CuO/ZnO sample to different concentrations of NO*_x_*; (*d*) gas response bar charts of 1 wt% In_2_O_3_–CuO/ZnO sample to 100 ppm different gases.

Figure 6*a* shows the gas response bar charts of different In_2_O_3_-doped concentration samples to different concentrations of NO*_x_*. The study found that the sensitivity of each sample decreased when NO*_x_* concentration decreased from 100 to 5 ppm. The results showed that In_2_O_3_ could significantly enhance the response of the materials to NO*_x_*, and the 1 wt% In_2_O_3_–CuO/ZnO sample had the best response to NO*_x_* among five prepared (0, 0.5, 1, 3, 6 wt%) In_2_O_3_–CuO/ZnO sensor samples in this study. At room temperature, the response and response time of the sensors with different In_2_O_3_ contents to 100 ppm NO*_x_* gas are shown in the electronic supplementary material, table S1.

Under the current operating conditions, the response–recovery curves of 1 wt% In_2_O_3_–CuO/ZnO sensor are shown in figure 6*b*. The resistance sharply decreased when the NO*_x_* gas was injected, and the resistance recovered to its initial value when the NO*_x_* gas was discharged. When the prepared sensor was in air, oxygen molecules were adsorbed on the surface of the sensor; the adsorbed oxygen captured electrons from the conductance band to produce negatively charged oxygen species. These chemisorbed oxygen species would act as surface acceptors, trapping electrons and increasing surface resistance of the sensor. When the NO*_x_* gas was injected, the NO*_x_* would react with the formed oxygen ions, which released the trapped electrons back to the sensor surfaces, and the resistance of the sensor was sharply decreased. It indicated that the prepared sensing material was a p-type semiconductor material.

Figure 6*c* shows the response and response time curve of the 1 wt% In_2_O_3_–CuO/ZnO sample to 100–1 ppm NO*_x_* at room temperature. When the NO*_x_* gas was injected into the sensing chamber, the resistance of the 1 wt% In_2_O_3_–CuO/ZnO sensor rapidly decreased, and reached the minimum resistance value in a short time. The 1 wt% In_2_O_3_–CuO/ZnO sensor exhibited a fast and reversible response to NO*_x_*, the response time being only 7 s to 100 ppm NO*_x_* which was much shorter than that reported in a lot of literature [26,39,41–43], and the corresponding response reached up to 82%. The responses were weakened with the reduction in the NO*_x_* concentration from 100 to 1 ppm, and the corresponding response time increased. It was related to the gas concentration, gas diffusion and adsorption on the surface of the nanomaterials. With decreasing of NO*_x_* concentration, the NO*_x_* gas partial pressure declined in the reaction chamber which led to gas phase reaction velocity being slower, and the response time increased. It is worth noting that the minimum detection limit of 1 wt% In_2_O_3_–CuO/ZnO gas sensor was only 1 ppm at room temperature, the corresponding response still reached 17% and the corresponding response time was 136 s.

In order to study the selectivity of 1 wt% In_2_O_3_–CuO/ZnO gas sensor, the responses of 1 wt% In_2_O_3_–CuO/ZnO sensor to different gases (NO*_x_*, NH_3_, O_2_, CO and H_2_) were measured at room temperature. The corresponding test results are shown in figure 6*d*. The 1 wt% In_2_O_3_–CuO/ZnO gas sensor had a relatively high response to NH_3_. When the NH_3_ concentration was 100 ppm, the response reached 48%. However, the response to NH_3_ of the sensor was also significantly lower than the response to the same concentration of NO*_x_*. The response of other gases (O_2_, CO and H_2_) was closer to 0%. The experimental results showed that the 1 wt% In_2_O_3_–CuO/ZnO gas sensor had good selectivity.

**Figure 7.**
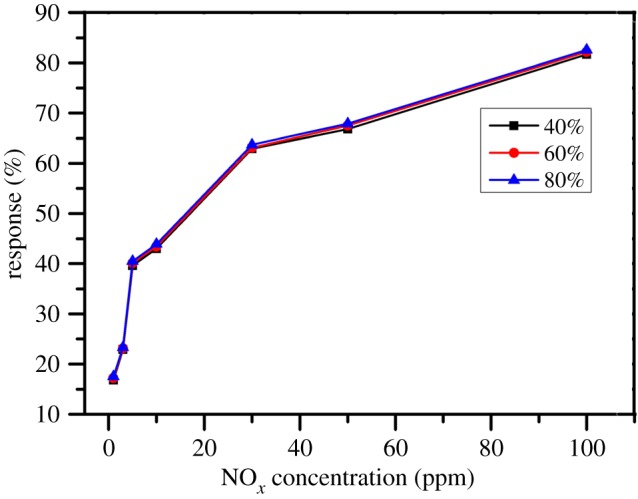
Influence of the humidity on the gas responses of 1 wt% In_2_O_3_–CuO/ZnO sample.

The humidity is one of the significant factors in the gas-sensing process, and the degree of humidity influences the sensing performance of oxide semiconductors. However, the humidity has little effect on NO*_x_* gas response in this study. The result is shown in figure 7.

**Figure 8.**
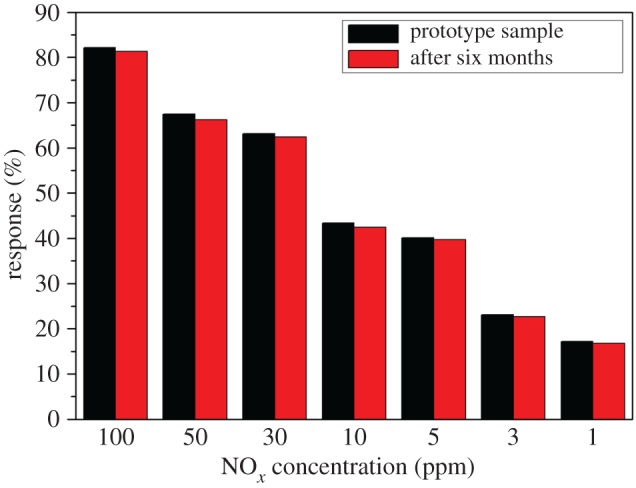
Stability bar charts of 1 wt% In_2_O_3_–CuO/ZnO sample.

Figure 8 shows the stability bar charts of 1 wt% In_2_O_3_–CuO/ZnO sample. The response of the prepared sample remained stable after six months. It indicated that the prepared 1 wt% In_2_O_3_–CuO/ZnO sample had good stability.

### 3.3. Discussion of sensing mechanism

The gas-sensing behaviour of ZnO results from the surface chemical reactions. The reactants are the chemisorbed oxygen species (O^−^, O_2_^−^ and O^2−^) and the adsorbed target gas molecules. These reactions lead to the resistance variation of the gas sensor. Generally, the n-type oxide semiconductor materials (such as ZnO) response mechanism involves the interaction between the detected gases and chemisorbed oxygen ions on the surface of the materials, leading to the resistance change of the sensor [44,45]. In air, the oxygen molecules adsorb on the surface of the materials, which can capture free electrons from the materials to form negative oxygen ions (O^2−^, O^−^ and O_2_^−^) at grain boundaries, and a thick space charge layer will be formed. The negative surface charges will generate a higher surface potential barrier, which leads to a high electrical resistance [46,47]. The process can be expressed as follows:

3.1 $${\text{O}}_{2}↔{{\text{O}}_{2}}^{-}↔{\text{O}}^{-}↔{\text{O}}^{2-}.$$

When the sensor is exposed in oxidizing NO*_x_* gas, the NO*_x_* gases attract the electrons from sensor materials, which can be attributed to their high electron affinity. The process leads to electrons of the sensor materials transfering to the surface NO*_x_*, and forming NO_2_^−^ (from NO_2_) and NO^−^ (from NO). The process traps electrons from the conduction band or donor level of materials, which leads to electron density reduction and the hole carrier density increase in the sensor. The hole carriers result in a decrease in resistance of the semiconductor layer. It will lead to a thinner space-charge layer, and reduce the potential barrier at the grain boundaries and cause a decrease in the sensor resistance [39]. The process can be expressed as follows:

3.2 $$\text{N}{\text{O}}_{2}+{\text{e}}^{-}\to \text{N}{{\text{O}}_{2}}^{-}/\text{NO}+{\text{e}}^{-}\to \text{N}{\text{O}}^{-}.$$

Also, the target gases NO_2_ and NO directly adsorb on the sensor, which react with O^−^ and generate bidentate NO_3_^−^ (s) and NO_2_^−^, and release the trapped electrons back into the conduction band. The process can be expressed as follows [48]:

3.3 $$\text{N}{\text{O}}_{2}+{\text{O}}^{-}\to \text{N}{{\text{O}}_{3}}^{-}/\text{NO + }{\text{O}}^{-}\to \text{N}{{\text{O}}_{2}}^{-}.$$

The band gap (*E*_g_) of ZnO is 3.4 eV, and its electron affinity (*c*) is 4.35 eV. The band gap of CuO is 1.35 eV, and its electron affinity is 4.07 eV. From a thermodynamic perspective, the electrons will transfer from the conduction band of CuO to ZnO, while the holes will oppositely migrate from the valence band of ZnO to CuO [49]. The process will increase the charge separation rate of electron–hole pairs under the bias potential, while the longer lifetime of electron–hole pairs of CuO/ZnO will be attributed to the formation of the p–n heterojunction. The p–n heterojunction may facilitate the adsorption of gas molecules. Thus, CuO/ZnO composite shows a higher sensing activity than pure ZnO, the pure ZnO has no gas response at room temperature. However, the formation of a p–n junction results in higher resistance [22].

The higher sensitivity of CuO/ZnO composite can be attributed to the electronic sensitization induced by CuO [50,51]. There are more active sites and more chemisorbed oxygen species on the surface of p-type semiconductors than n-type semiconductors [51,52]. The modified CuO nanostructures can act as a strong acceptor of electrons from ZnO [50]. The Fermi energy levels of n-type ZnO and p-type CuO are equalized which is due to the charge transfer [53,54]. The introduction of CuO film on the surface of the porous ZnO increases the carrier density of the ZnO, which results in the electron redistribution between the ZnO and the CuO [55]. In the air, oxygen molecules capture electrons from CuO, and chemisorbed oxygen species are formed on its surface [56]. Meanwhile, electrons transfer from ZnO to CuO, which causes the formation of an electron depletion layer (EDL) extending into ZnO [51]. The EDL significantly narrows the conducting part and dominates the conductivity of CuO/ZnO [57,58]. The decoration of CuO promotes the chemisorption of oxygen species and the chemical reactions on the surface of CuO/ZnO [51,59]. This leads to an enhancement in the sensitivity of CuO/ZnO composite [60].

In addition, high specific surface area of sensing material usually has positive effects on the sensing response [61]. The porous structure helps to increase the gas-sensing performance. The porous structure provides abundant pores, which is more beneficial to diffusion of gas molecules [62,63]. Moreover, it also provides more active sites, and improves the kinetics of chemical reactions on the surface [63]. This also results in enhanced sensing performance. Furthermore, there would be more oxygen species chemisorbed on the surface of CuO film structures due to its large surface to volume ratio [64]. Thus, the sensitivity of the CuO/ZnO composite is further enhanced. In this study, CuO is introduced onto the surface of ordered mesoporous zinc oxide by electrodeposition after chemical deposition method. The active site density is improved by the advantages of porous and large specific surface area of mesoporous materials. CuO is dispersed on the porous surface of the mesoporous ZnO matrix material to provide an effective gas diffusion channel for CuO contacting with the gas. The provided multiple active sites make NO*_x_* easier to react with adsorbed oxygen ions on the surface, which increases gas sensitivity performance.

On the surface of the prepared CuO/ZnO composite, the heterogeneous structure can be formed between CuO and ZnO by ultrasonic method, and the gas-sensitive properties can be enhanced by In_2_O_3_. Generally, the n–n (or p–n) type heterojunction-based interfacial barriers between In_2_O_3_ and ZnO (or CuO) nanocrystals are another mechanism accounting for the enhanced sensing performance [45,65–67]. In this study, the doping content is very little which forms discontinuity phase. A possible gas-sensing mechanism is as follows: in the band of interfaces, there is a spike at the In_2_O_3_ side along with a notch at the host side. The discontinuity band sets barriers for electrons in both conduction band and valence band. The heterojunction interfacial barriers can strongly modify the charge transport behaviours of carriers because the carrier concentration varies exponentially with the barrier height. Further studies are required to determine the mechanism through which the In_2_O_3_-doped semiconductor interfacial barriers promote the sensing performance towards gases [68]. Also, the catalysis of the In_2_O_3_ should not be overlooked. The catalytic activity of In_2_O_3_ accelerates the dissociation of oxygen molecules, and causes a spillover of the adsorbed oxygen ions on the surface of 1% In_2_O_3_–CuO/ZnO composite. More adsorbed oxygen ions provide more sensing activity sites and shows high response [45,69–71]. In this study, the doped quantity In_2_O_3_ is very little, the p–n heterojunction or n–n/p–p homo-type heterojunction cannot be formed, and the In_2_O_3_ only plays a role of sensitizer to sensitize the surface reaction. With the In_2_O_3_ content increasing, the sensitization is enhanced. But, the sensitized mechanism is changed when the doped In_2_O_3_ quantity exceeded the limit, which can be explained with heterojunction mechanism, and the response of the sensor will be the lower when the In_2_O_3_ content is not enough to form the continuous heterojunction.

**Figure 9.**
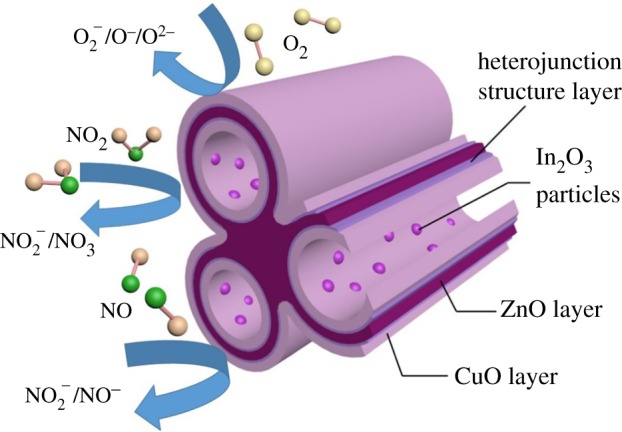
A schematic diagram of sensing mechanism of 1 wt% In_2_O_3_–CuO/ZnO sensor.

In this study, the remarkable gas-sensing performance of the prepared 1 wt% In_2_O_3_–CuO/ZnO sensor may be for the following reasons. First, the fluffy porous structure is helpful for gas diffusion and surface reaction, which lead to the sensor exhibiting faster response. Second, n–p heterojunction between CuO and ZnO plays an important role in the enhancement of the sensor response and selectively. The CuO has a lower Fermi level than ZnO, and ZnO would receive electrons from CuO, leading to the formation of an accumulation layer at the CuO/ZnO interface. The increase in electrons on the surface of ZnO is able to facilitate adsorption of oxygen molecules and a decrease in resistance. Third, the doping of In_2_O_3_ is also helpful for remarkable gas-sensing performance of 1 wt% In_2_O_3_–CuO/ZnO composite. The reasons include electron interactions between In_2_O_3_ and ZnO, the catalytic activity of In_2_O_3_ accelerates the dissociation of oxygen molecules, and causes a spillover of the adsorbed oxygen ions on the surface of 1 wt% In_2_O_3_–CuO/ZnO composite. More adsorbed oxygen ions provide more sensing sites, and show high response. The mechanism is intuitively explained in figure 9.

## 4. Conclusion

An ordered mesoporous ZnO nanomaterial has been synthesized by a template method. The prepared porous ZnO nanomaterial is used as matrix to prepare a porous Cu film–ZnO composite by the electroplating after chemical plating method, and finally a CuO film-decorated ordered porous ZnO composite is obtained by a high-temperature oxidation method. Then, In_2_O_3_ is loaded into the prepared CuO/ZnO by an ultrasonic-assisted method, affording an In_2_O_3_–CuO/ZnO composite. The prepared 1 wt% In_2_O_3_–CuO/ZnO composite shows the best gas-sensing performance, in which the In_2_O_3_ doping content is only 1 wt%. For 1 wt% In_2_O_3_–CuO/ZnO sample, its response time is only 7 s to 100 ppm NO*_x_*, and the corresponding response reaches 82%. It is worth noting that the minimum detection limit of 1 wt% In_2_O_3_–CuO/ZnO gas sensor was only 1 ppm at room temperature, the corresponding response still reached 17% and the corresponding response time was 136 s. The response of the 1 wt% In_2_O_3_–CuO/ZnO gas sensor is much higher, the minimum detection limit is much lower, the selectivity and stability are much better and the corresponding response time is much shorter at room temperature than that reported in a lot of literature.

## Supplementary Material

Response and response time to 100 ppm NOx gas of the sensors with the different In2O3 contents at room temperature.

## Data accessibility

All relevant data are within the paper and the electronic supplementary material files.
