# Supplementary material for: Study on room temperature gas-sensing performance of CuO film-decorated ordered porous ZnO composite by In2O3 sensitization
Source: R Soc Open Sci. 2018 Feb 14;5(2):171788. doi: 10.1098/rsos.171788 (PMC5830776; doi:10.1098/rsos.171788)
Supplement: Response and response time to 100 ppm NOx gas of the sensors with the different In2O3 contents at room temperature. [file rsos171788supp1.docx]

**Supporting information**

**Study on room temperature gas sensing performance of CuO film decorative ordered porous ZnO composite by In_2_O_3_ sensibilization**

Tian-tian Li^a^, Na Bao^a^, Ai-fang Geng ^a, *^, Hui Yu^a, *^, Ying Yang^a^，Xiang-ting Dong^a^

(a. School of Chemistry ＆ Environmental Engineering, Changchun University of Science and Technology, Changchun 130022, P. R. China)

**Table S1.** Response and response time to 100 ppm NO_x_ gas of the sensors

with the different In_2_O_3_ contents at room temperature.

| **In_2_O_3_ contents (wt %)** | 0 | 0.5 | 1 | 3 | 6 |
| --- | --- | --- | --- | --- | --- |
| **Response (%)** | 35.1 | 66.3 | 82.2 | 67.5 | 72.7 |
| **Response Time (s)** | 72 | 27 | 7 | 26 | 23 |
